# The Unenterprising British Practitioner

**Published:** 1909-08-07

**Authors:** 


New Series. No. 128, Vol. V. [No. 1200, Vol. XLVI.] SATURDAY, AUGUST 7, 1909.

### THE UNENTERPRISING BRITISH PRACTITIONER.

Whatever may be the scientific attainments of our professional brethren in foreign lands, we cannot resist the conclusion that the average British medical practitioner, in town or country or colony, is superior in many vital and essential respects to his contemporaries in foreign countries. This, of course, is merely an expression of opinion which cannot be verified by any instrument of precision or supported by evidence equal to the test of severe and formal cross-examination. Nevertheless, we have strong support for this conviction in the series of articles, as yet uncompleted, upon foreign medical schools and methods, which has been appearing at intervals for many months in our columns, from the pen of our Special Commissioner. Even a casual study of these reports upon the great Continental and American medical centres cannot fail to confirm the idea or awaken the suspicion, that—whatever may be the opportunities and facilities offered by the leading foreign schools of medicine, and whatever the attainments and achievements of their professors and *alumni*—the system of medical education in London and Edinburgh and Dublin and Cambridge, for example, despite its anomalies and its drawbacks, continues to turn out practitioners of medicine more capable, more self-reliant, and more dependable, than the average of their colleagues conducting general practices in the towns and villages of other countries. This, we must repeat, is an assertion, an expression of opinion, and no more. But so is the well-accepted statement that the English Public School, in spite of its inferiority in methods of teaching, remains the admiration and the despair of every astute and wide-minded visitor to our country from the educational establishments of foreign lands.

The point is that the end is more important than the means. It is almost an axiom that Germany is the home and centre of pure scientific and of medico-scientific research, that the German temperament and frame of mind transcends our own by far in zeal and practical capacity for patient and productive scientific investigation. Nevertheless, there are strong grounds for believing that the British practitioner of medicine, educated and brought up as he is upon haphazard and illogical lines, has a finer and firmer professional *common sense* than the generality of his Teutonic and other Continental colleagues. Within the ranks of the practitioners of our paradoxical islands we seem to see an abiding healthy scepticism of those new-fledged, half-hatched synthetic remedies and frothy hypothetical methods of treatment which (to judge by contemporary Continental medical journalism) are a perpetual source of excitement and speculation and fatuous experimentation to a large number of other European and trans-Atlantic medical men.

It will not we think be denied even by the youngest amongst us that a thorough knowledge and experience of the old and well-tried remedies is the only proper foundation and excuse for a reasonable inquiry into the virtues of any one of the innumerable new synthetic drugs whose dubious claims to notice and trial are pressed so urgently and so boisterously upon every British practitioner whose name and address is accessible to the German manufacturer or his English representative. It can scarcely be doubted that amongst our Continental colleagues there exists a friendlier and a more credulous attitude towards the blatant pretensions of unscrupulous drug houses, and towards the scarcely less fatuous and unprofitable outpourings of pseudo-scientific investigators, than is found in general among British practitioners. It is not fair, of course, to judge the whole medical profession of a nation by those among its members who allow their names to bolster up the latest unscrupulous additions to the commercial Pharmacopœia. But where there is much smoke there must at least be a little fire, and it is neither unkind nor unreasonable to apply the phrase *ex pede Herculem* to the profession of any country wherein the drug manufacturers exploit their wares upon the reckless, brazen, and unscrupulous recommendation of hordes of "venal physicians." It may be that the strict code of professional morality and manners enforced upon us in this country by a stern, unbending Medical Council is the sole check upon our tendencies to overt self-advertisement, commercialism, and unethical practices of every sort: but we think not. Black sheep undoubtedly disgrace every walk of life, and those of our number who would if they dared besmirch our calling are certainly kept in check by the fear of penal arraignment before the official guardians of our professional honour and dignity in these islands. But we believe that our antiquated and contradictory British methods of general and medical education are not only favourable to the development of sound practical common sense and ability, but also to the development of that pride in our profession and solicitude for its good name, which is its only sure protection from the ever-growing modern canker of commercialism and corruption. Thus it is that we can look with equanimity upon the exasperatingly dull and apathetic attitude of the average British practitioner towards the experiments, the achievements, and the absurdities of Continental scientific medicine, which seems at first glance so unenterprising and so grossly unprogressive.

